# Thalassosterol, a New Cytotoxic Aromatase Inhibitor Ergosterol Derivative from the Red Sea Seagrass *Thalassodendron ciliatum*

**DOI:** 10.3390/md18070354

**Published:** 2020-07-08

**Authors:** Reda F. A. Abdelhameed, Eman S. Habib, Marwa S. Goda, John Refaat Fahim, Hashem A. Hassanean, Enas E. Eltamany, Amany K. Ibrahim, Asmaa M. AboulMagd, Shaimaa Fayez, Adel M. Abd El-kader, Tarfah Al-Warhi, Gerhard Bringmann, Safwat A. Ahmed, Usama Ramadan Abdelmohsen

**Affiliations:** 1 Department of Pharmacognosy, Faculty of Pharmacy, Suez Canal University, Ismailia 41522, Egypt; omarreda_70@yahoo.com (R.F.A.A.); emansnd@yahoo.com (E.S.H.); marwa_saeed@pharm.suez.edu.eg (M.S.G.); Drhashemhassanean2020@pharm.suez.edu.eg (H.A.H.); enastamany@gmail.com (E.E.E.); am_kamal66@yahoo.com (A.K.I.); 2 Department of Pharmacognosy, Faculty of Pharmacy, Minia University, Minia 61519, Egypt; john.mcconnell@lancet.com (J.R.F.); usama.ramadan@mu.edu.eg (U.R.A.); 3 Pharmaceutical Chemistry Department, Faculty of Pharmacy, Nahda University, BeniSuef 62513, Egypt; asmaa_owis@yahoo.com; 4 Institute of Organic Chemistry, University of Würzburg, Am Hubland, 97074 Würzburg, Germany; shaimaa.seaf@uni-wuerzburg.de; 5 Department of Pharmacognosy, Faculty of Pharmacy, Ain-Shams University, Cairo 11566, Egypt; 6 Department of Pharmacognosy, Faculty of Pharmacy, Deraya University, New Minia 61111, Egypt; ad_cognosy@yahoo.com; 7 Department of Pharmacognosy, Faculty of Pharmacy, Al-Azhar University, Assiut 71524, Egypt; 8 Department of Chemistry, College of Science, Princess Nourah bint Abdulrahman University, Riyadh 13414, Saudi Arabia; tarfah-w@hotmail.com

**Keywords:** cytotoxic activity, ergosterol derivative, metabolic analysis, docking studies, seagrass, *Thalassodendron ciliatum*

## Abstract

*Thalassodendron ciliatum* (Forssk.) Den Hartog is a seagrass belonging to the plant family Cymodoceaceae with ubiquitous phytoconstituents and important pharmacological potential, including antioxidant, antiviral, and cytotoxic activities. In this work, a new ergosterol derivative named thalassosterol (**1**) was isolated from the methanolic extract of *T. ciliatum* growing in the Red Sea, along with two known first-reported sterols, namely ergosterol (**2**) and stigmasterol (**3**), using different chromatographic techniques. The structure of the new compound was established based on 1D and 2D NMR spectroscopy and high-resolution mass spectrometry (HR-MS) and by comparison with the literature data. The new ergosterol derivative showed significant in vitro antiproliferative potential against the human cervical cancer cell line (HeLa) and human breast cancer (MCF-7) cell lines, with IC_50_ values of 8.12 and 14.24 µM, respectively. In addition, docking studies on the new sterol **1** explained the possible binding interactions with an aromatase enzyme; this inhibition is beneficial in both cervical and breast cancer therapy. A metabolic analysis of the crude extract of *T. ciliatum* using liquid chromatography combined with high-resolution electrospray ionization mass spectrometry (LC-ESI-HR-MS) revealed the presence of an array of phenolic compounds, sterols and ceramides, as well as di- and triglycerides.

## 1. Introduction

Seagrasses are marine flowering plants that grow underwater along temperate and tropical coastlines, providing shelter or food for other marine organisms. They are important components of the near-shore ecosystem and are used as biological indicators of environmental quality [1]. In East Africa, seagrasses are used as fertilizers and medicinally for the management of fever and skin diseases [2]. *Thalassodendron ciliatum* (Forssk.) Den Hartog (Family Cymodoceaceae) is a sub-tidal or shallow-depositional seagrass species that is generally found in extensive and monotonous meadows. This sickle-leaved cymodocea, commonly known as "Majani kumbi", grows in the Red Sea, the western Indian Ocean, and the Indo-Pacific region [3]. *T. ciliatum* has both horizontal and vertical rhizomes, with a cluster of leaves at the top of each living stem [4]. The leaves of *T. ciliatum* are characterized by the presence of many tannin-containing cells and are rich in phenolic constituents [5]. Pharmacological studies showed that *T. ciliatum* possesses antioxidant, antiviral, and cytotoxic activities, which are highly correlated to its flavonoid content [6,7,8]. Several flavonoids were reported from *T. ciliatum* such as quercetin 3-*O*-*β*-d-xylopyranoside, asebotin, 3-hydroxyasebotin, rutin, and racemic catechin [6]. Recently, a new diglyceride ester and asebotin were isolated from *T. ciliatum*, showing antiviral activities against the H5N1 virus through inhibition of the virus titre by 67.26% and 53.81%, respectively [7]. A new dihydrochalcone diglycoside was also identified from *T. ciliatum*, exhibiting anti-influenza A virus activity [9]. In 2018, our group isolated a cytotoxic phytosphingosine-type ceramide from *T. ciliatum* of the Red Sea in addition to different sterols with anti-inflammatory effects [10]. Gas chromatography coupled to mass spectrometry analysis (GC-MS coupling) was performed for identification of the lipoidal matter of the *n*-hexane fraction of *T. ciliatum*, revealing the presence of saturated and unsaturated long-chain fatty acids, such as tetradecanoic acid, eicosanoic acid, 9,12-hexadecadienoic acid, and 8,11,14- eicosatrienoic acid, in addition to other volatile compounds, as well as 1-heneicosanol, 2,6-bis (1,1-dimethylethyl)phenol, and 1-tridecanol [11]. Some genera of Cymodoceaceae showed different steroidal profiles, among them compounds like cholesterol, *ß*-sitosterol, stigmasterol, campesterol, and 22,23-dihydrobrassicasterol. Four 3-ketosteroids with a 24-ethyl cholestane side chain were also isolated from *Cymodocea nodosa* [12,13]. A comparative study on five seagrasses of different families revealed a high percentage of stigmasterol and *ß*-sitosterol in both *Cymodocea serrulate* and *Halodule uninervis* [14]. Our previous study was the only one that reported on the isolation of different steroids from *T. ciliatum* with anti-inflammatory effects, including 7*β*-hydroxy cholesterol, 7*β*-hydroxysitosterol, stigmasterol glucoside, and *β*-sitosterol glucoside [10]. Therefore, we herein continue our chemical and biological investigation on *T. ciliatum* growing in the Red Sea, along with high-resolution electrospray ionization mass spectrometry (LC-ESI-HR-MS)-assisted metabolic profiling of this important seagrass. 

## 2. Results and Discussion

### 2.1. Metabolic Profiling

**Figure 1:**
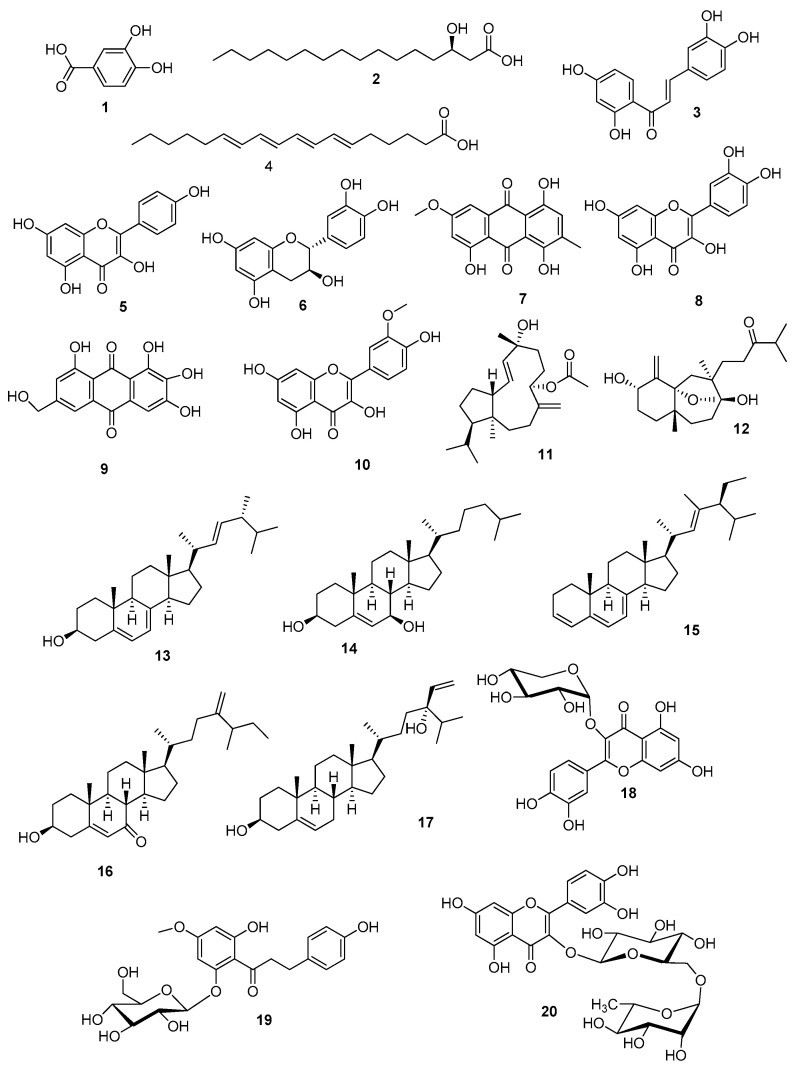
Chemical structures of the detected metabolites listed in Table 1.

Metabolic profiling of the crude extract of *T. ciliatum* using the LC-ESI-HR-MS technique (Figures S1 and S2) revealed the presence of a broad variety of metabolites such as flavonoids, chalcones, phenolic acids, sterols, fatty acids, anthraquinones, and terpenoids. The LC-ESI-HR-MS profiling for the rapid identification of natural metabolites resulted in the annotation of 20 compounds identified by comparison of their data, particularly their accurate masses, with those from some databases, e.g., the Dictionary of Natural Products (DNP) and the Metabolite and Chemical Entity (METLIN) database, as shown in Figure 1. Mass accuracy was calculated as [measured mass-expected mass/expected mass] × 10^6^ and expressed in parts per million (ppm) error [15]. The herein-characterized metabolites of *T. ciliatum* were found to be in accordance with those obtained in previous phytochemical studies, which reported the isolation of several phenolic compounds and sterols [6,10]. From Table 1, protocatechuic acid, butein, kaempferol, catechin, 1,4,5-trihydroxy-7-methoxy-3-methyl anthraquinone, quercetin, 2-ω-dihydroxy emodin, isorhamnetin, quercetin-3-*O*-*β*-d-xylopyranoside, asebotin, and rutin were reported to have antioxidant activity that can protect from cardiovascular diseases, liver damage, and proliferation of abnormal cells [6,16,17,18,19,20]. Butein showed an aromatase inhibition activity that can be effective in breast cancer treatment [21]. A long-chain polyunsaturated fatty acid, namely 6*E*,8*E*,10*E*,12*E*-octadecatetraenoic acid, as well as the diterpene linearol, exhibited antimicrobial and anticancer activities [22,23]. Likewise, both sphaerollane I and ergosterol inhibited proliferation of various malignant cell lines [24,25]. Linearol and rutin were reported to have antioxidant, cytotoxic and antiviral activities [18,23]. It is worth noting that the aforementioned antioxidant, antiviral, and cytotoxic activities of the crude extract of *T. ciliatum* may be correlated to the activities of the identified metabolites.

### 2.2. Identification of the Isolated Metabolites

**Figure 2:**
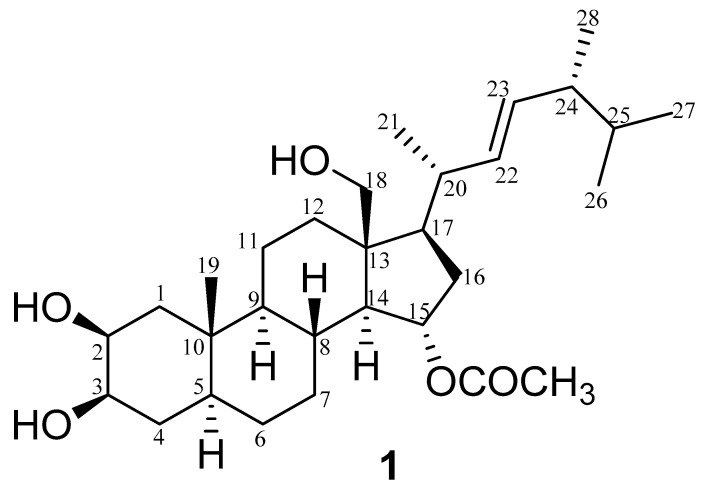
Chemical structure of the new ergosterol derivative **1** (2*β*,18-dihydroxy-15*α*-acetoxy-5,6,7,8-tetrahydroergosterol), named thalassosterol.

**Figure 3:**
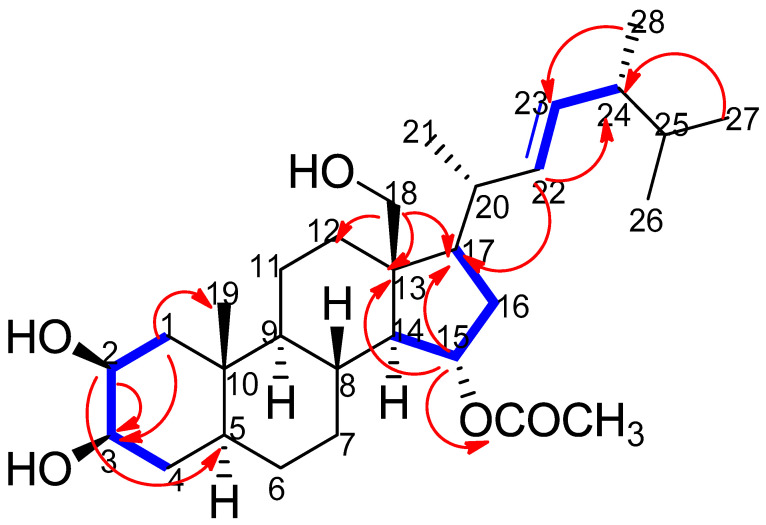
Key ^1^H-^1^H COSY (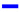) and Heteronuclear Multiple Bond Correlation (HMBC) (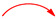) interactions in thalassosterol (**1**), (2*β*,18-dihydroxy-15*α*-acetoxy-5,6,7,8-tetrahydroergosterol). Blue bold line represents ^1^H-^1^H COSY correlations between H1/H2, H2/H3, H3/H4, H14/H15, H15/H16, H16/H17, H22/H23 and H23/H24, while red single-head arrow represents HMBC correlations between H1/C19, H1/C3, H2/C3, H2/C5, H15/C13, H15/C17, H15/CO, H18/C12, H18/C17, H18/C13, H22/C17, H22/C24, H27/C24 and H28/C23.

Compound **1** (Figure 2) was obtained as a white powder and its molecular formula was determined by ESI-HR-MS as C_30_H_49_O_5_ (found at *m/z* 489.3573 [M − H]^−^ (Figure S3), calculated for 489.3579), indicating the presence of six degrees of unsaturation in the molecule. The NMR spectral data of compound **1** (Table 2) (Figures S4–S7) also closely matched those reported in the literature for ergostane-type steroids [27,37], showing signals characteristic of four secondary methyl groups resonating at *δ*_H_ 1.06 (CH_3_-21), 0.92 (CH_3_-28), 0.877 (CH_3_-27), and 0.873 (CH_3_-26), as well as one tertiary methyl group at *δ*_H_ 1.03 (CH_3_-19), along with their carbon resonances at *δ*_C_ 19.9, 18.4, 22.9, 23.1, and 14.1, respectively. The two geminally coupled (*J* = 12.2 Hz) one-proton doublets at *δ*_H_ 3.75 and 3.65, and the corresponding oxygenated methylene carbon at *δ*_C_ 60.6 were indicative of the presence of a hydroxy group at C-18, which was corroborated by the observed Heteronuclear Multiple Bond Correlation (HMBC) correlations of H_2_-18 with C-12, C-13, and C-17 (Figure 3) (Figure S8). Additionally, the NMR data of compound **1** displayed two one-proton doublets of doublets at *δ*_H_ 5.12 (*J* = 15.2, 6.5 Hz) and 5.23 (*J* = 15.2, 7.2 Hz), ascribable to the olefinic protons H-22 and H-23, together with their corresponding methine carbons at *δ*_C_ 137.5 and 133.1, respectively. 

The position of this double bond between C-22 and C-23 was also supported by the obtained Heteronuclear Multiple Bond Correlation (HMBC) cross peaks of H-22/C-17 and H-22/C-24, as well as by the proton/proton Correlation Spectroscopy (^1^H-^1^H COSY) of H-22/H-23 and H-23/H-24 (Figure 3 and Figure S9), whereas the large coupling constant (15.2 Hz) between H-22 and H-23 typically indicated a trans-configuration of that double bond. Moreover, the ^1^H NMR signals at *δ*_H_ 4.71 (1H, br. d, *J* = 2.2 Hz) and 4.74 (1H, br. d, *J* = 2.2 Hz) were attributed to H-2 and H-3, respectively, suggesting that the hydroxylation in ring A of this sterol should be at C-2 and C-3, which was also confirmed by Heteronuclear Single Quantum Correlation (HSQC). These assignments were also substantiated with the aid of ^1^H-^1^H COSY, HSQC, and HMBC analyses (Figure 3).

**Figure 4:**
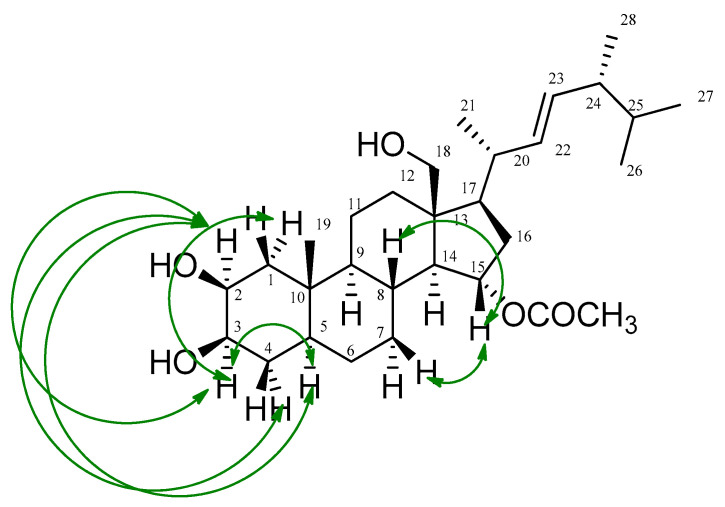
Key Nuclear Overhauser Effect Spectroscopy (NOESY) (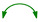) correlations of thalassosterol (**1**), (2*β*,18-dihydroxy-15*α*-acetoxy-5,6,7,8-tetrahydroergosterol). Green double-head arrow represents NOESY correlations between H2/H3, H2/H5, H2/H4a, H3/H1a, H3/H2, H3/H5, H15/H7b and H15/H8.

The *β*-configuration of the hydroxy groups at C-2 and C-3 was clearly deduced from the observed chemical shifts of both carbons (*δ*_C_ 75.9 and 76.3, respectively) [38], as well as through the Nuclear Overhauser Effect Spectroscopy (NOESY) correlations observed between H-2/H-3, H-2/H-5, H-2/H-4a, H-3/H-1a, H-3/H-2, and H-3/H-5 (Figure 4 and Figure S10). On the other hand, the three-proton singlet at *δ*_H_ 2.0, along with its corresponding carbon resonating at *δ*_C_ 21.2 and the quaternary carbonyl signal at *δ*_C_ 172.6 were consistent with the presence of an acetoxy group, which was unambiguously allocated at C-15 based on the marked downfield shift of both H-15 (*δ*_H_ 4.98) and C-15 (*δ*_C_ 74.3) in comparison with ergosterol (**2**) and other related ergostane derivatives [25,37]. The noticed HMBC correlation of H-15 with the carbonyl carbon of this acetoxy group, in addition to its three-bond connectivities to both C-13 and C-17, were also in good agreement with the acetylation of compound **1** at C-15, while the observed NOESY correlations between H-15 and both H-7b and H-8 verified the *α*-orientation of that acetoxy group (Figure 4). Based on the above-mentioned assignments, which were totally supported by the DEPT-135, ^1^H-^1^H COSY, HSQC, HMBC, and NOESY experiments, compound **1** was identified as 2*β*,18-dihydroxy-15*α*-acetoxy-5,6,7,8-tetrahydroergosterol, or, in other words, as (2*β*,3*β*-dihydroxy-13*β*-hydroxymethyl-10*β*-methyl-17*β*-(1,4,5-trimethyl-hex-2*E*-enyl)-hexadecahydro-cyclopenta[α]phenanthren-15*α*-yl acetate) (Figure 2). To the best of our knowledge, this molecule is a new compound, henceforth named thalassosterol. 

**Figure 5:**
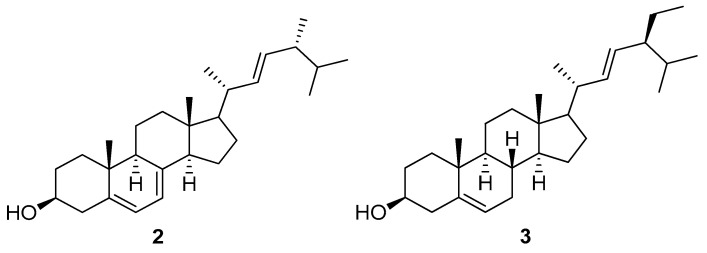
Chemical structures of the known metabolites ergosterol (**2**) and stigmasterol (**3**) isolated from *T. ciliatum.*

Compounds **2** and **3** (Figure 5) were identified as the known steroids ergosterol (**2**) and stigmasterol (**3**), respectively, by comparing their spectral data with those reported in the literature [25,37,39,40]. Both compounds were also detected in the metabolic analysis of *T. ciliatum*. It is worth mentioning that it is the first time to report on the isolation of both ergosterol and stigmasterol from the seagrass *T. ciliatum*. 

### 2.3. Docking Study

**Figure 6:**
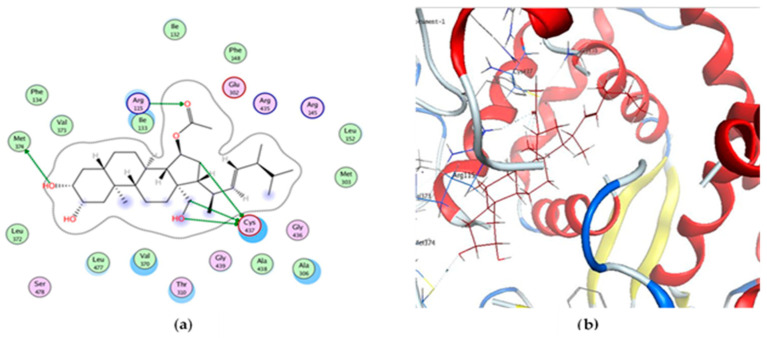
(**a**) The 2D caption of thalassosterol (**1**), (2*β*,18-dihydroxy-15*α*-acetoxy-5,6,7,8-tetrahydroergosterol), binding to the active site of aromatase. (**b**) Binding pattern of compound **1** colored by element into the receptor binding site showing five interactions (dotted lines).

It can be observed generally through looking at the best score poses that binding of the new ergosterol, thalassosterol (**1**), at the active site is similar to that of the endogenous aromatase substrate, exemestane [41]. The binding interaction shows that the steroidal nucleus, overlapped with the hydrophobic environment of the binding pocket with ring D, is oriented towards the Met 374 residue and the *β*-face positioned towards the heme moiety. It can be clearly noticed that, while the hydroxy group of the ring A engages one hydrogen bond donor with the backbone amide of Met 374 as reported (3.82 Å) [42], the C15-keto oxygen atom of ring D acts as a hydrogen bond acceptor from Arg 115 (3.12 Å). Moreover, three extra hydrogen bond donors were involved via the interaction with Cys 417 residue with binding interaction score −8.219 kcal/mol (Figure 6). It is worth to mention that the H-bond interaction of the targeted new compound with Met 374 represents the selectivity of the latter with the active site of the aromatase. Moreover, the binding interactions of the target ergosterol derivative with different amino acids of the binding site of the aromatase did not only confirm the selectivity but also the possible binding interactions when compared with exemestane.

### 2.4. In Vitro Antiproliferative Activity

The SRB assay is a rapid, sensitive, and inexpensive method for screening for antitumor activities of chemical agents against different malignant cell lines [43]. The antitumor effects of the isolated compounds were assessed by determination of the concentration required for 50% of growth inhibition (IC_50_). The cytotoxic potentials of the new compound **1** and of doxorubicin as a positive control are shown in Table 3 against different malignant cell lines, including the human cervical cancer cell line (HeLa), a human breast cancer cell line (MCF-7), and a human liver cancer cell line (HepG2). The efficacy of compound **1** follows the order of HeLa > MCF-7 > HepG2 malignant cell lines. The IC_50_ of compound **1** is 8.13 ± 0.21 µM (mean ± SD) against the HeLa cell line and this result is closely related to the one obtained with doxorubicin as a positive control (Table 3). Moreover, compound **1** showed a weak antiproliferative activity on cells of the normal Vero cell line (> 90 µM).

According to the US NCI (National Cancer Institute) plant screening program guidelines, a crude extract is considered to have in vitro antiproliferative activity if the IC_50_ value after incubation between 48 and 72 h is smaller than 20 µg/mL, and less than 4 µg/mL for pure compounds [44]. Based on these NCI guidelines, the new compound **1** exhibited a high cytotoxic potential against the HeLa cell line, with an IC_50_ value of 8.13 ± 0.21 µM (less than 8.17 µM, equivalent to 4 µg/mL of the US NCI guidelines). On the other hand, compound **1** showed a weak cytotoxic activity against the HepG2 cell line, with an IC_50_ value of 48.64 ± 0.22 µM. Moreover, it was found to have a moderate cytotoxic activity against the breast cancer cell line MCF-7, with an IC_50_ value of 14.26 ± 0.40 µM when compared with doxorubicin as a positive control (8.65 ± 0.03 µM). This cytotoxic activity against the mentioned types of estrogen-responsive cancers, cervical cancer and breast cancer [41] was confirmed by the previously discussed docking study by blocking the aromatase enzyme that synthesizes estrogens.

## 3. Materials and Methods 

### 3.1. Plant Material

The seagrass *T. ciliatum* was collected from Sharm El Sheikh at the Egyptian Red Sea, then air-dried and stored at a low temperature (‒24 °C) until further processing. The plant was identified by Dr. Tarek Temraz, Marine Science Department, Faculty of Science, Suez Canal University, Ismailia, Egypt. A voucher sample (no. SAA-41) was deposited in the herbarium section of Pharmacognosy Department, Faculty of Pharmacy, Suez Canal University, Ismailia, Egypt.

### 3.2. General Experimental Procedures

^1^H and ^13^C NMR spectra were obtained with a Bruker Avance III HD 400 spectrometer operating at 400 MHz for ^1^H and 100 MHz for ^13^C. Both ^1^H and ^13^C NMR chemical shifts are expressed in *δ* values in regard to the solvent peaks *δ*_H_ 3.3 and *δ*_C_ 49 ppm for CD_3_OD, and coupling constants are given in Hertz (Hz). Thin layer chromatography (TLC) analysis was carried out on aluminum-backed plates pre-coated with silica gel F_254_ (20 × 20 cm; 200 µm; 60 Å (Merck^™^, Darmstadt, Germany), while silica gel 60/230–400 µm mesh size (Whatman^™^, Sanford, ME, USA) was used for column chromatography. Sephadex^®^ LH-20 (Sigma Aldrich, Bremen, Germany), and reversed-phase octadecyl silica (ODS) gel (YMC, Kyoto, Japan) were also utilized.

### 3.3. Extraction and Isolation

The air-dried material of *T. ciliatum* (90 g) was ground and extracted with a 1:1 mixture of CH_2_Cl_2_/MeOH (2 L × 3) at room temperature. The combined extracts were concentrated under vacuum to afford a dark-green residue (Tc, 21 g), which was then chromatographed over an open silica gel column using *n*-hexane/ethyl acetate (EtOAc) as the eluent (95:5‒0:100) and EtOAc/methanol (90:10‒50:50), with gradient elution giving seven fractions, Tc-A~Tc-G. The second fraction eluted using 25% EtOAc in hexane, Tc-B, was concentrated to afford a green residue (5 g) and was purified on silica gel using hexane/EtOAc (95:5) giving nine subfractions, Tc-B-1~Tc-B-9. Among them, subfractions Tc-B-2~Tc-B-4, having similar TLC patterns, were combined (Tc-B-1′, 90 mg) and re-chromatographed on silica gel using hexane-EtOAc (95:5) giving four sub-subfractions, Tc-B-1′-1~Tc-B-1′-4. One of the resulting sub-subfractions, Tc-B-1′-2, afforded compound **3** (11 mg). Another sub-subfraction, Tc-B-1′-3 (30 mg), was also applied to a Sephadex LH-20 column and eluted with CHCl_3_-MeOH (1:1) to afford compounds **1** (14 mg, white powder) and **2** (16 mg). Compound **1** was finally purified on an open ODS column using MeOH/H_2_O (8:2).

### 3.4. Metabolic Profiling

The metabolic study was performed using LC-ESI-HR-MS for dereplication purposes according to the literature [45]. An aliquot of 10 μL of the methanolic extract of *T. ciliatum* (1 mg/mL) was injected to an Accela HPLC (Thermo Fisher Scientific, Bremen, Germany) using an ACE C18 column of 75 mm × 3 mm having 5 μm internal diameter (Hichrome Limited, Reading, UK) and equipped with an Accela UV-visible detector. This compartment was coupled to an Exactive (Orbitrap) mass spectrometer (Thermo Fisher Scientific, Bremen, Germany). The gradient elution was done using purified water (total organic carbon 20 ppb) and acetonitrile; each containing 0.1% formic acid with a flow rate of 300 μL/min at ambient temperature. The gradient elution technique was assessed by increasing the concentration of acetonitrile from 10% to 100% within 30 min, followed by an isocratic period of 5 min and then reducing the concentration of acetonitrile to 10% within 1 min. ESI-HR-MS analysis was performed in both positive and negative ionization modes, with a spray voltage of 4.5 kV and a capillary temperature of 320 °C. The ESI-MS mass range was set at *m/z* 100–2000 using in-source collision-induced dissociation (CID) mechanism and *m/z* 50–1000. The raw HR-MS data were imported and analyzed using MZmine 2.12. Excel macros were also employed to dereplicate each *m/z* ion peak with metabolites in the database, using the retention time and an *m/z* threshold of ± 5 ppm, to attain the tentative identification of the compounds. A chemotaxonomic filter was applied to the obtained hits in order to limit the number of identities per metabolite and to only include the relevant ones. The compounds were identified by a comparison of their data, particularly their accurate masses, with those from some databases, e.g. DNP and METLIN. The retention time, *m/z*, molecular weight, molecular formula, MZmine ID, name, and biological source were determined.

### 3.5. Modeling Study on the Binding Between the New Ergosterol Derivative 1 and the Aromatase Binding Site

Molecular Operating Environment (MOE) was adopted for docking calculations. The structures of the compounds were generated by ChemDraw Ultra 11.0. Molecular docking calculations were applied on human placental aromatase (PDB code 3S7S, http://www.rcsb.org/pdb/home/home.do). Since the aromatase enzyme is complexed with the steroid exemestane as an inhibitor, this model was chosen for steroidal scaffold inhibitors. Docking simulation was done on the test compounds with the following protocol. Structure arrangement process was used to revise the protein errors, and a reasonable protein structure was set up on default rules on MOE. Finally, the Gasteiger methodology was used to calculate the partial charges of the protein [46]. The ligands were protonated and the correction of atom and bond types were defined, hydrogen atoms were added, and finally minimization was performed (MMFF94x, gradient: 0.01).

The default Triangle Matcher placement method was selected for docking. The GBVI/WSA dG scoring function that determines the free energy of binding of the ligand from a given pose, was chosen to rank the final poses. The ligand complex with the enzyme having the lowest S-score was selected. The redocking of ligand with its target revealed an RMSD of 0.98 Å, which confirmed that the ligand binds to the same pocket and assured the dependability of parameters of docking.

### 3.6. In Vitro Antiproliferative Assay

#### 3.6.1. In Vitro Cell Culture

Human breast adenocarcinoma (MCF-7), cervical cancer (HeLa), and liver carcinoma (HepG2) cell lines were purchased from the American Type Culture Collection (ATCC, Alexandria, MN, USA). The tumor cell lines were preserved at the National Cancer Institute, Cairo, Egypt, by serial sub-culturing. The cells were sub-cultured on RPMI 1640 medium supplemented with 1% penicillin/streptomycin and 10% fetal bovine serum [47].

#### 3.6.2. Sulforhodamine B Assay

The antiproliferative activity was determined by the sulforhodamine B (SRB) assay. SRB is a fluorescent aminoanthracene dye with two sulfonic acid groups that bind to the amino groups of intracellular proteins under mildly acidic conditions to provide a sensitive index of cellular protein content. The test was performed as previously described in detail [48,49]. Cells were seeded in 96-well microtiter plates at an initial concentration and left for 24 h to attach to the plates. Then, the three isolated compounds were dissolved in dimethyl sulfoxide (DMSO) and added at different concentrations of 0, 5, 12.5, 25, and 50 µg/mL. After incubation for 48 h, 50 μL of 10% trichloroacetic acid were added and incubated for 60 min at 4 °C to fix the attached cells. The plates were washed and stained with 50 μL of 0.4% SRB dissolved in 1% acetic acid for 30 min at room temperature. The plates were then air-dried, and the dye was solubilized with 100 μL/well of 10 M tris base of pH 10.5 (AppliChem^®^, Darmstadt, Germany). Optical density (OD) was measured spectrophotometrically at 570 nm using an ELISA microplate reader (Sunrise Tecan reader, Crailsheim, Germany). The experiment was repeated three times and IC_50_ values (concentration that causes 50% decrease in cell viability) were calculated. Doxorubicin served as a positive control at the same concentration range.

## 4. Conclusions

From the CH_2_Cl_2_/MeOH extract of the Red Sea grass *T. ciliatum*, a new ergosterol derivative, compound **1**, was isolated. It was identified as 2*β*,18-dihydroxy-15*α*-acetoxy-5,6,7,8-tetrahydroergosterol and named thalassosterol. The new compound displayed a significant cytotoxic activity against cervical (HeLa) and breast cancer cell lines (MCF-7), with IC_50_ values of 8.12 and 14.24 µM, respectively. In addition, docking studies with compound **1** explained how it works as an aromatase inhibitor, serving a protocol of cervical and breast cancer treatment. LC-MS based metabolic analysis of *T. ciliatum* furthermore revealed the presence of phenolic compounds, sterols, ceramides, and di- and triglycerides. Therefore, the presented work highlights *T. ciliatum* as an important marine source of bioactive secondary metabolites that should attract further chemical and pharmacological investigation. 

## Figures and Tables

**Table 1:** Metabolic analysis of a crude extract of *Thallasodendron ciliatum.*

	Polarity Mode	Retention Time (min)	MZmine ID	*m/z* *	Measured Mass	Expected Mass	Mass Error (ppm)	Name	Molecular Formula **	Source	Ref.
1	positive	1.51	9930	155.0347	154.0274	154.0266	5.2	Protocatechuic acid	C_7_H_6_O_4_	*Allium cepa*	[26]
2	negative	11.29	382	271.2269	272.2342	272.2351	−3.3	3*R*-Hydroxypalmitic acid	C_16_H_32_O_3_	*Saccharomycopsis* sp.	[27]
3	positive	5.07	9019	273.0748	272.0675	272.0685	−3.7	Butein	C_15_H_12_O_5_	*Dalbergia odorifera*	[17]
4	positive	5.63	9699	277.2153	276.2080	276.2089	−3.3	6*E*,8*E*,10*E*,12*E*-Octadecatetraenoic acid	C_18_H_28_O_2_	*Anadyomene stellata*	[28]
5	negative	4.46	3937	285.0393	286.0466	286.0477	−3.8	Kaempferol	C_15_H_10_O_6_	*Fragaria chiloensis*	[29]
6	negative	2.18	3954	289.0705	290.0778	290.0790	−4.1	Catechin	C_15_H_14_O_6_	*T. ciliatum*	[6]
7	negative	5.43	4767	299.0551	300.0623	300.0634	−3.7	1,4,5-Trihydroxy-7-methoxy-3-methylanthraquinone	C_16_H_12_O_6_	*Chaetomium globosum*	[30]
8	negative	5.07	4942	301.0341	302.0414	302.0427	−4.3	Quercetin	C_15_H_10_O_7_	*Fragaria chiloensis*	[29]
9	positive	3.37	9298	303.0503	302.0430	302.0427	0.9	2-ω-Dihydroxyemodin	C_15_H_10_O_7_	*Aspergillus nidulans*	[31]
10	negative	4.76	4129	315.0497	316.0570	316.0583	−4.1	Isorhamnetin	C_16_H_12_O_7_	*Stigma maydis*	[32]
11	negative	13.90	4382	347.2581	348.2654	348.2664	−2.9	Sphaerollane I	C_22_H_36_O_3_	*Sphaerococs coronopifolis*	[33]
12	positive	8.66	12289	353.2706	352.2634	352.2614	5.7	Linearol	C_21_H_36_O_4_	*Sideritis condensata*	[23]
13	positive	12.71	9842	397.3452	396.3379	396.3392	−3.3	Ergosterol (**2**)	C_28_H_44_O	*Ganoderma lucidum*	[25]
14	positive	13.60	10050	403.3528	402.3456	402.3498	−10.4	7*β*-Hydroxycholesterol	C_27_H_46_O_2_	*T. ciliatum*	[10]
15	positive	12.49	8715	407.3677	406.3604	406.3600	0.9	23-Methylstigmasta-3*Z*,5*Z*,7*Z*,22*E*-tetraene	C_30_H_46_	*Suillus luteus*	[34]
16	positive	10.46	8941	427.3572	426.3499	426.3498	0.2	26-Methylergosta-5,24(28)-diene-7-one-3-ol	C_29_H_46_O_2_	*Geodia japonica*	[35]
17	positive	15.43	320	429.3732	428.3659	428.3654	1.2	24*R*-Stigmasta-5,28-diene-3*β*,24 -diol	C_29_H_48_O_2_	*Sargassum fusiforme*	[36]
18	negative	3.37	4110	433.0765	434.0838	434.0849	−2.5	Quercetin-3-*O*-*β*-D-xylopyranoside	C_20_H_18_O_11_	*T. ciliatum*	[6]
19	positive	6.10	705	451.1642	450.1570	450.1526	9.8	Asebotin	C_22_H_26_O_10_	*T. ciliatum*	[6]
20	negative	2.33	5311	609.1466	610.1539	610.1534	0.8	Rutin	C_27_H_30_O_16_	*T. ciliatum*	[6]

* *m/z* is expressed in negative or positive formula; ** Molecular formula is expressed in a neutral formula.

**Table 2:** ^1^H (400 MHz) and ^13^C (100 MHz) NMR spectroscopic data of compound **1** (CD_3_OD, *δ* in ppm, *J* in Hz).

No.	*δ*_H_	*δ*_C_
**1**	1.42, 2.09, m	39.1
**2**	4.72, br. d (*J* = 2.2)	76.0
**3**	4.75, br. d (*J* = 2.2)	76.3
**4**	1.60, 1.80, m	30.4
**5**	1.59, m	40.2
**6**	1.25, 1.50, m	28.9
**7**	0.96, 1.59, m	32.3
**8**	1.74, m	32.4
**9**	0.83, m	56.8
**10**	----	36.5
**11**	1.37, 1.56, m	21.8
**12**	0.96, 2.62, m	36.2
**13**	---	48.8
**14**	1.20, m	59.8
**15**	4.99, m	74.4
**16**	1.30, 2.46, m	39.9
**17**	1.14, m	58.1
**18**	3.65, d (*J* = 12.2) 3.75 d (*J* = 12.2)	60.6
**19**	1.03, br. s	14.2
**20**	1.72, m	36.6
**21**	1.06, d (*J* = 6.4)	19.9
**22**	5.15, dd (*J* = 15.2, 6.5)	137.5
**23**	5.21, dd (*J* = 15.2, 7.2)	133.2
**24**	1.84, m	44.4
**25**	1.51, m	29.2
**26**	0.873, d (*J* = 6.64)	23.2
**27**	0.877, d (*J* = 6.64)	23.0
**28**	0.92, d (*J* = 6.8)	18.4
**-CO-CH_3_**	2.00, s	21.2
**-CO-CH_3_**	----	172.6

**Table 3:** IC_50_ values (µM) of thalassosterol (**1**) and doxorubicin against different human cell lines, HeLa, human liver cancer cell line (HepG2), and human breast cancer cell line (MCF-7).

	Human Cancer Cell Lines		
	HeLa IC50 (µM)	HepG2 IC50 (µM)	MCF-7 IC50 (µM)
**Thalassosterol (1)**	8.13 ± 0.21 *	48.64 ± 0.22 *	14.26 ± 0.40 *
**Doxorubicin**	6.77 ± 0.07	9.02 ± 0.06	8.65 ± 0.03

Human cervical cancer cell line (HeLa), human liver cancer cell line (HepG2), human breast cancer cell line (MCF-7). * Significantly different from doxorubicin as the positive control. Each data point represents the mean ± SD of three independent experiments (significant differences at *p* < 0.05).

## Supplementary Materials

The following are available online at https://www.mdpi.com/1660-3397/18/7/354/s1, Figures S1–S2: LC-ESI-HR-MS of the crude extract (positive and negative modes), Figures S3–S10: ESI-HR-MS, 1H NMR, 13C NMR, DEPT-135, HSQC, HMBC, COSY and NOESY of compound 1.
