# Supplementary material for: Thalassosterol, a New Cytotoxic Aromatase Inhibitor Ergosterol Derivative from the Red Sea Seagrass Thalassodendron ciliatum
Source: Mar Drugs. 2020 Jul 8;18(7):354. doi: 10.3390/md18070354 (PMC7401251; doi:10.3390/md18070354)
Supplement: Supplementary file 1 [file marinedrugs-18-00354-s001.pdf]

Thalassosterol, a New Cytotoxic Aromatase Inhibitor Ergosterol Derivative from the Red Sea Grass *Thalassodendron ciliatum*

Reda F. A. Abdelhameed [1,†], Eman S. Habib [1,†], Marwa S. Goda [1], John Refaat Fahim [2], Hashem A. Hassanean [1], Enas E. Eltamany [1], Amany K. Ibrahim [1], Asmaa M. AboulMagd [3], Shaimaa Fayez [4,5], Adel M. Abd El-kader [6,7], Tarfah Al-Warhi [8], Gerhard Bringmann [4,*], Safwat A. Ahmed [1,*] and Usama Ramadan Abdelmohsen [2,6]

[1] Department of Pharmacognosy, Faculty of Pharmacy, Suez Canal University, 41522 Ismailia, Egypt
[2] Department of Pharmacognosy, Faculty of Pharmacy, Minia University, 61519 Minia, Egypt
[3] Pharmaceutical Chemistry Department, Faculty of Pharmacy, Nahda University, 62513 BeniSuef, Egypt
[4] Institute of Organic Chemistry, University of Würzburg, Am Hubland, 97074 Würzburg, Germany
[5] Department of Pharmacognosy, Faculty of Pharmacy, Ain-Shams University, 11566 Cairo, Egypt
[6] Department of Pharmacognosy, Faculty of Pharmacy, Deraya University, 61111 New Minia, Egypt
[7] Department of Pharmacognosy, Faculty of Pharmacy, Al-Azhar University, Assiut 71524, Egypt
[8] Department of Chemistry, College of Science, Princess Nourah bint Abdulrahman University, Riyadh, Saudi Arabia
† Equal contributions
* Correspondence: bringmann@chemie.uni-wuerzburg.de (G.B.); safwat_aa@yahoo.com (S.A.A.); Tel.: (+49) 0931-318 5323 (G.B.); (+20) 010-92638387 (S.A.A.),

Figure No.	Title	Page
**Figure S1.**	Chromatogram of LC-ESI-HR-MS analysis of the crude extract of *Thalassodendron ciliatum* (positive mode)	3
**Figure S2.**	Chromatogram of LC-ESI-HR-MS analysis of the crude extract of *Thalassodendron ciliatum* (negative mode)	4
**Figure S3.**	Chromatogram of ESI-HR-MS of compound **1**	5
**Figure S4.**	^{1}H NMR spectral data of compound **1**	6
**Figure S5.**	^{13}C NMR spectral data of compound **1**	7
**Figure S6.**	DEPT-135 NMR spectral data of compound **1**	8
**Figure S7.**	HSQC chart of compound **1**	9
**Figure S8.**	HMBC chart of compound **1**	10
**Figure S9.**	COSY chart of compound **1**	11
**Figure S10.**	NOESY chart of compound **1**	12

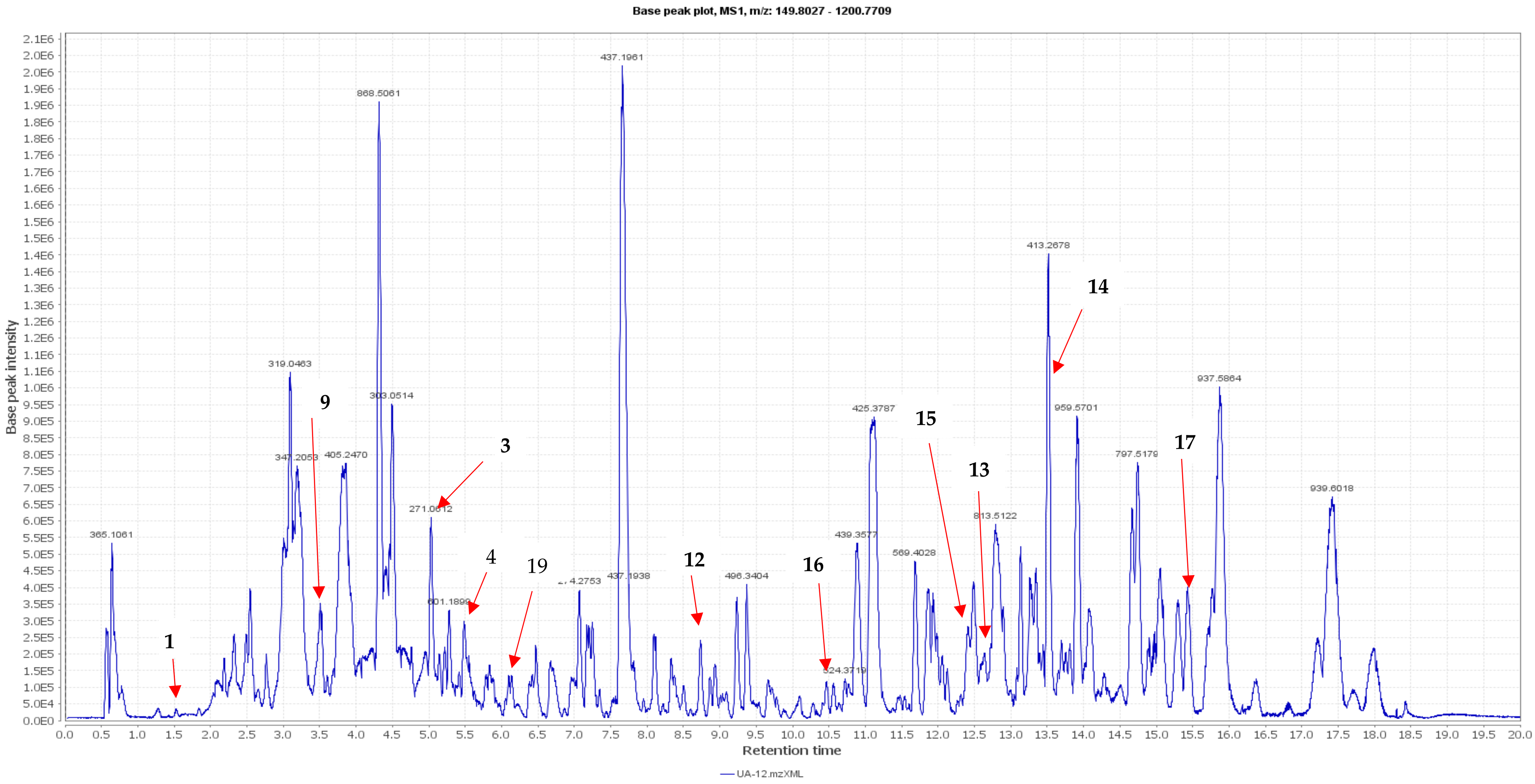

Figure S1. Chromatogram of LC-ESI-HR-MS analysis of crude extract of *Thalassodendron ciliatum* (positive mode).

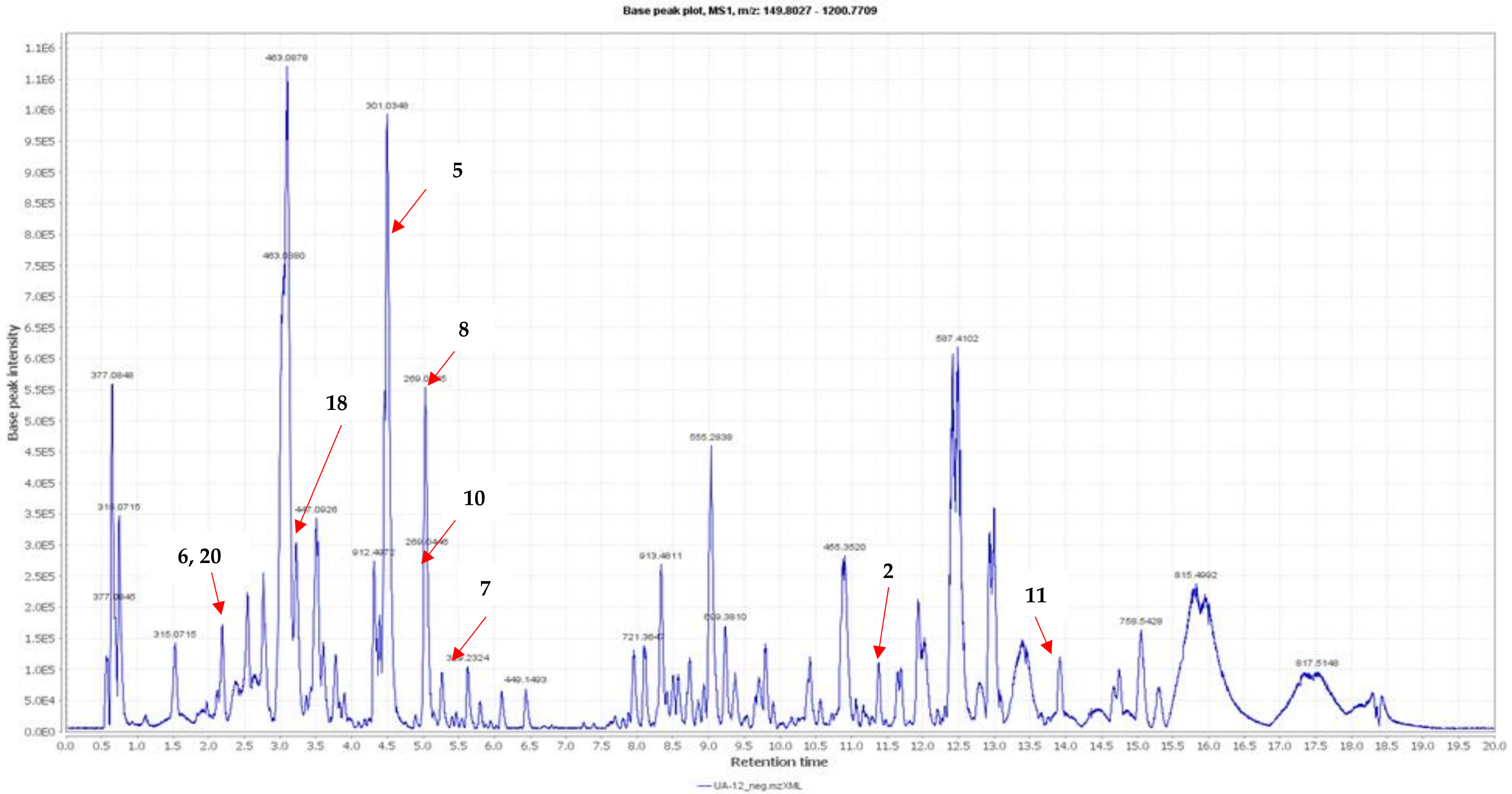

Figure S2. Chromatogram of LC-ESI-HR-MS analysis of crude extract of *Thalassodendron ciliatum* (negative mode).

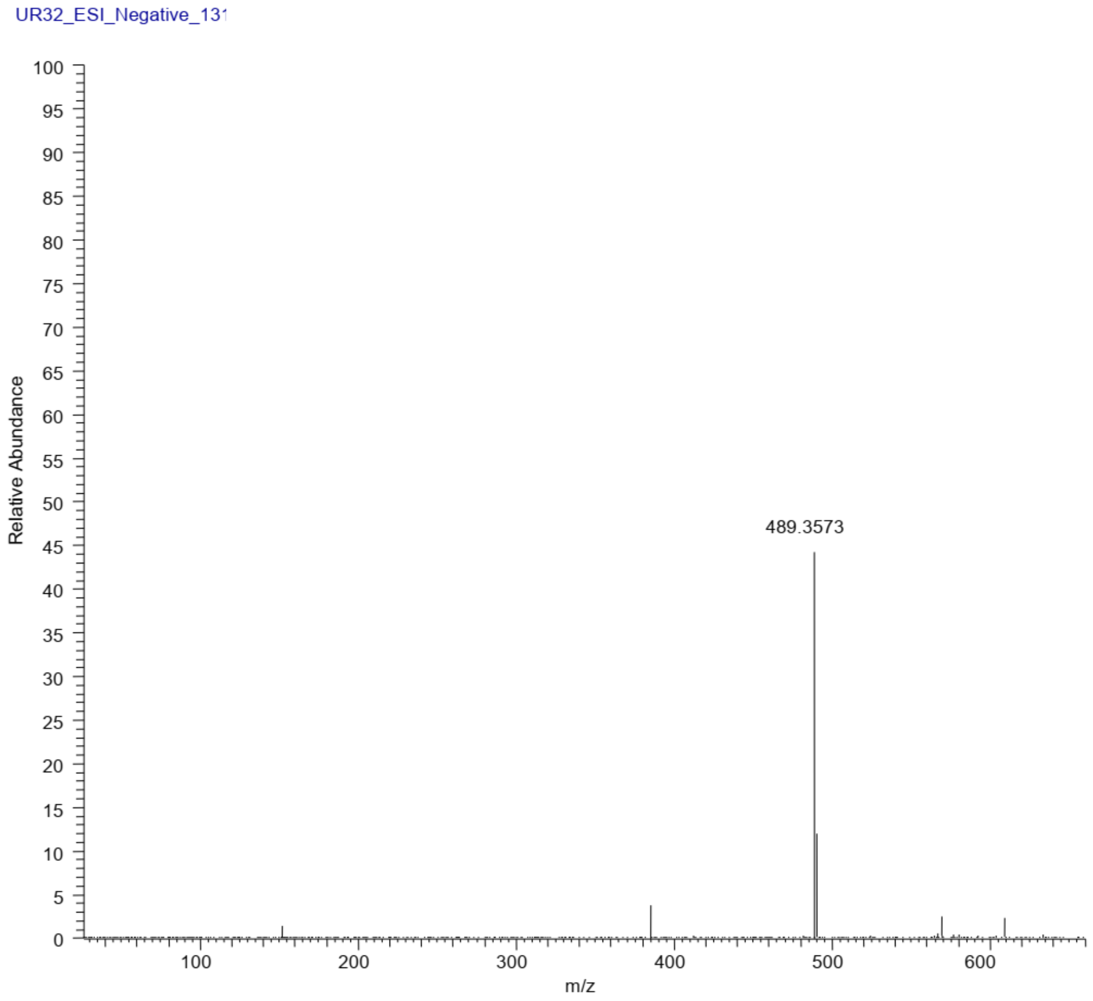

Figure S3. Chromatogram of ESI-HR-MS of thalassosterol (**1**, 2*β*,18-dihydroxy-15*α*-acetoxy-5,6,7,8-tetrahydroergosterol).

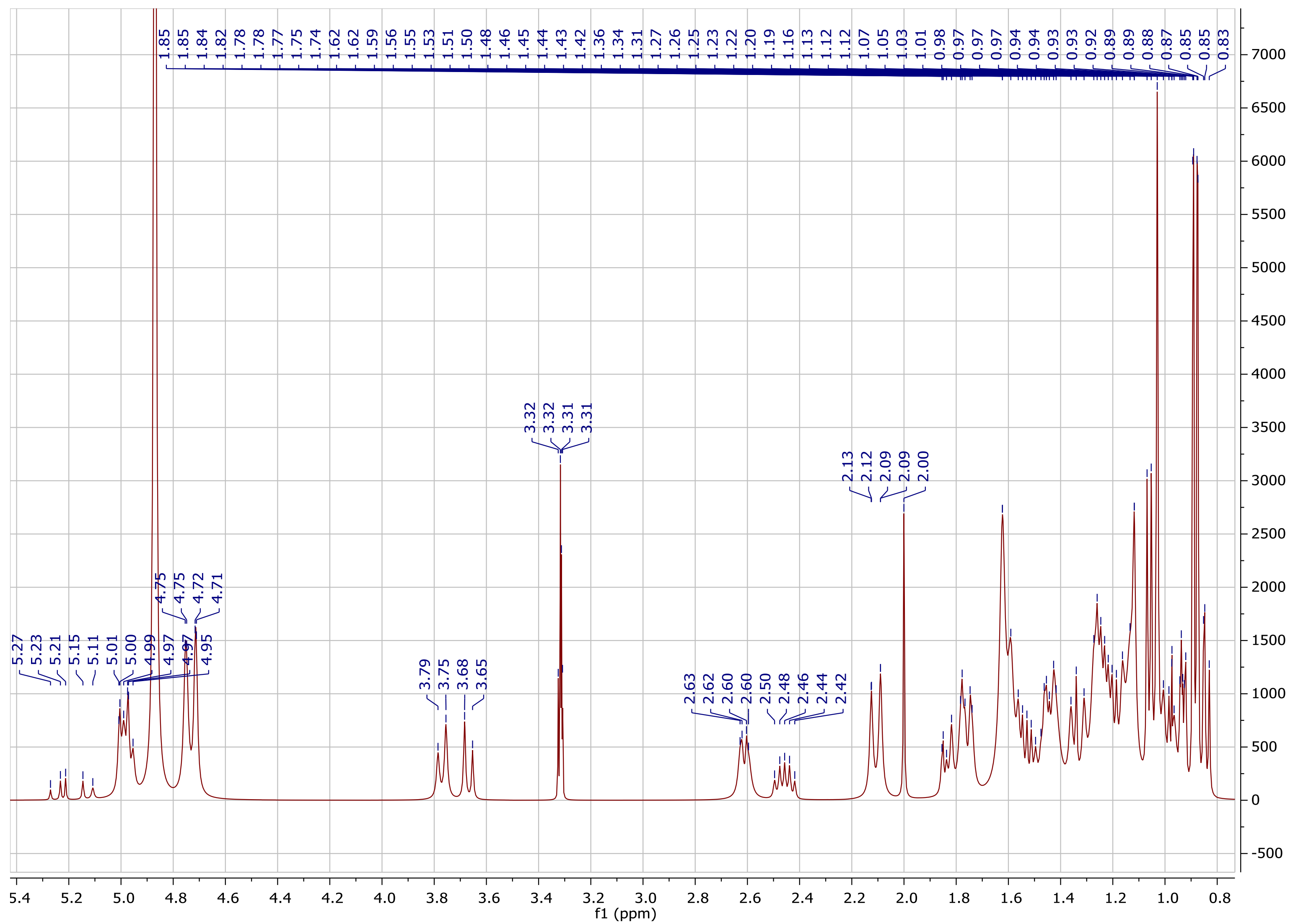

Figure S4. ^{1}H NMR spectral data of thalassosterol (**1**, 2β,18-dihydroxy-15α-acetoxy-5,6,7,8-tetrahydroergosterol).

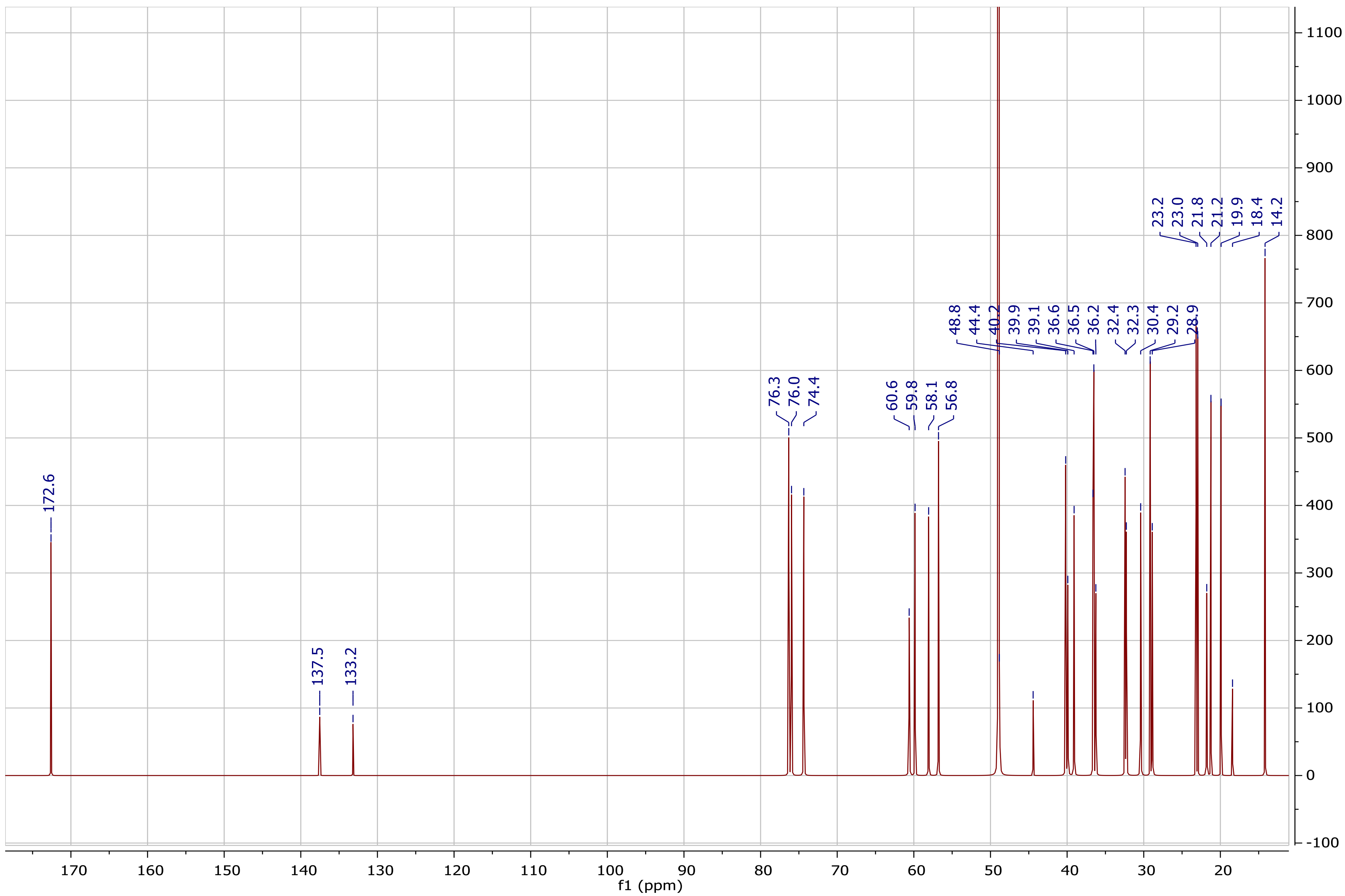

Figure S5. ^{13}C NMR spectral data of thalassosterol (**1**, 2*β*,18-dihydroxy-15*α*-acetoxy-5,6,7,8-tetrahydroergosterol).

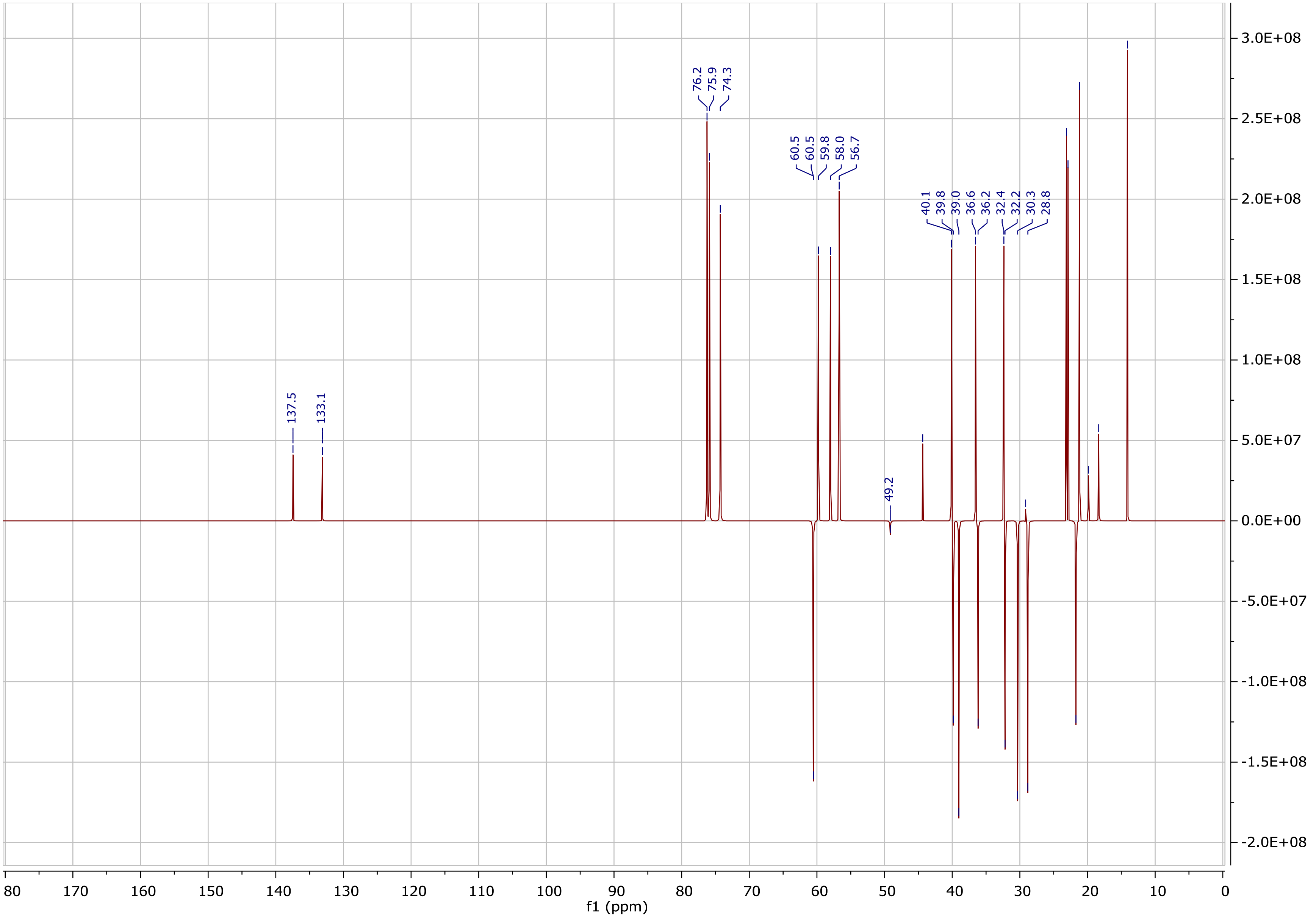

Figure S6. DEPT-135 spectral data of thalassosterol (**1**, 2β,18-dihydroxy-15α-acetoxy-5,6,7,8-tetrahydroergosterol).

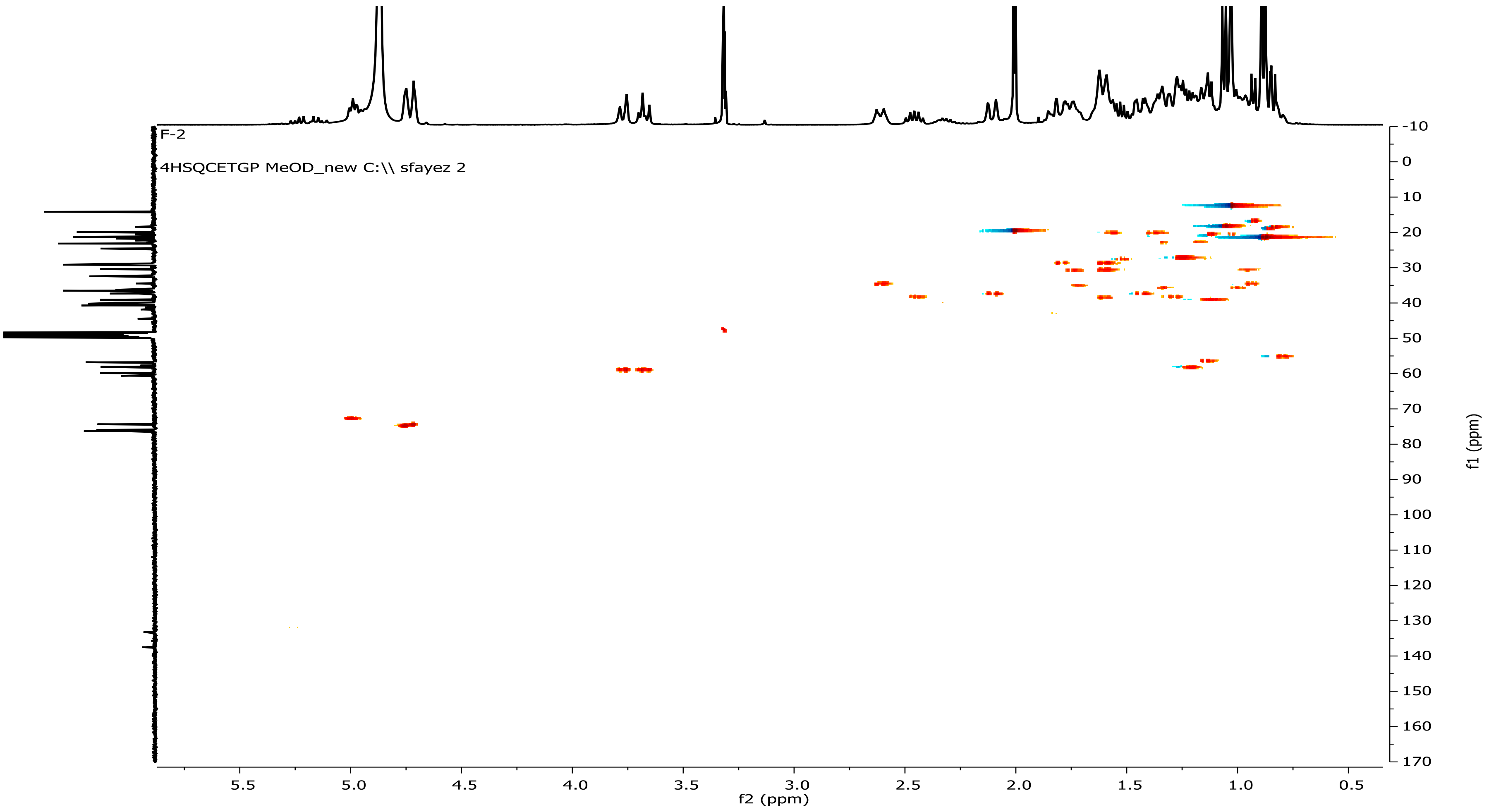

Figure S7. HSQC chart of thalassosterol (**1**), (2β,18-dihydroxy-15α-acetoxy-5,6,7,8-tetrahydroergosterol).

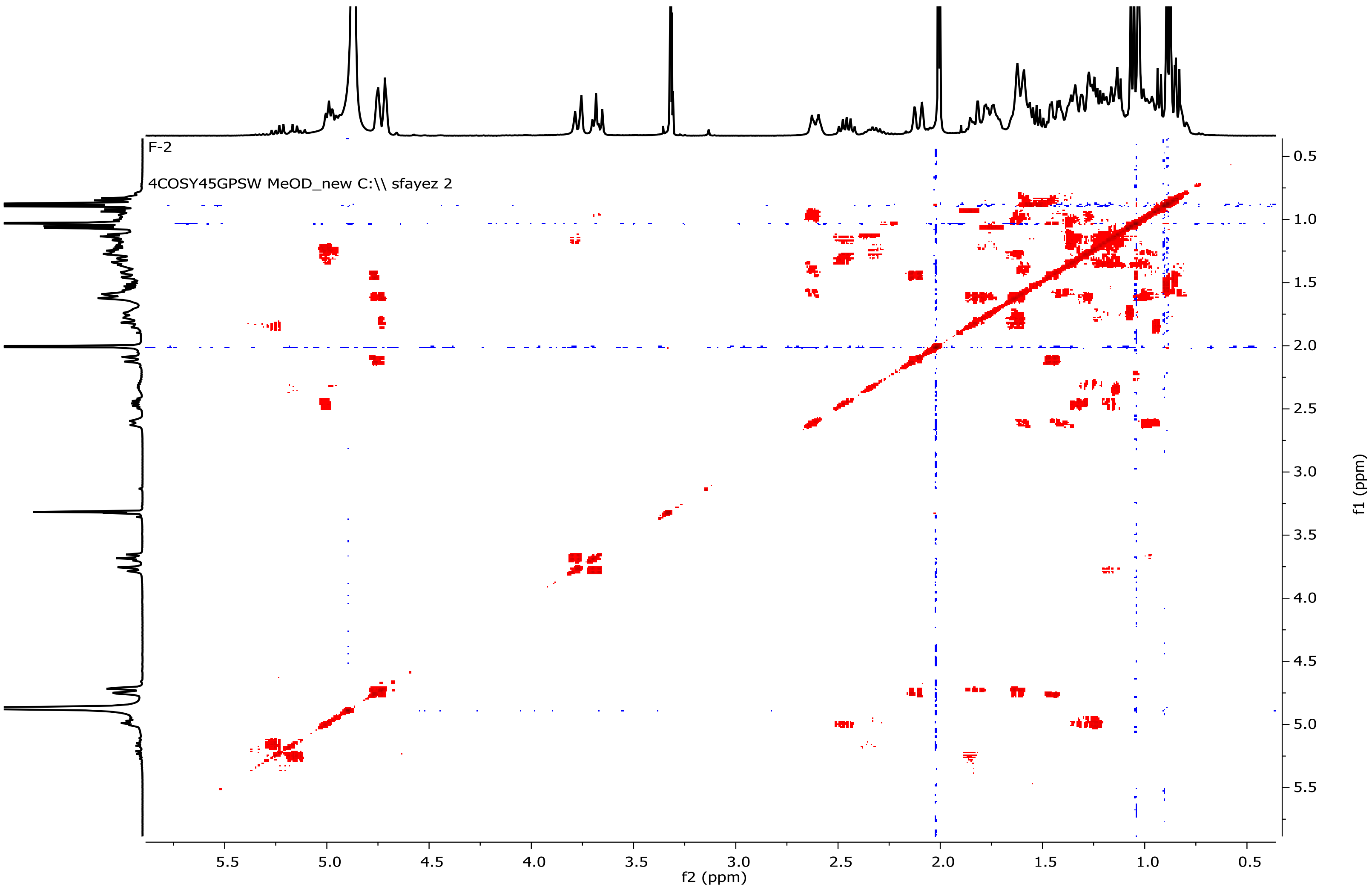

Figure S8. COSY chart of thalassosterol (**1**, 2β,18-dihydroxy-15α-acetoxy-5,6,7,8-tetrahydroergosterol).

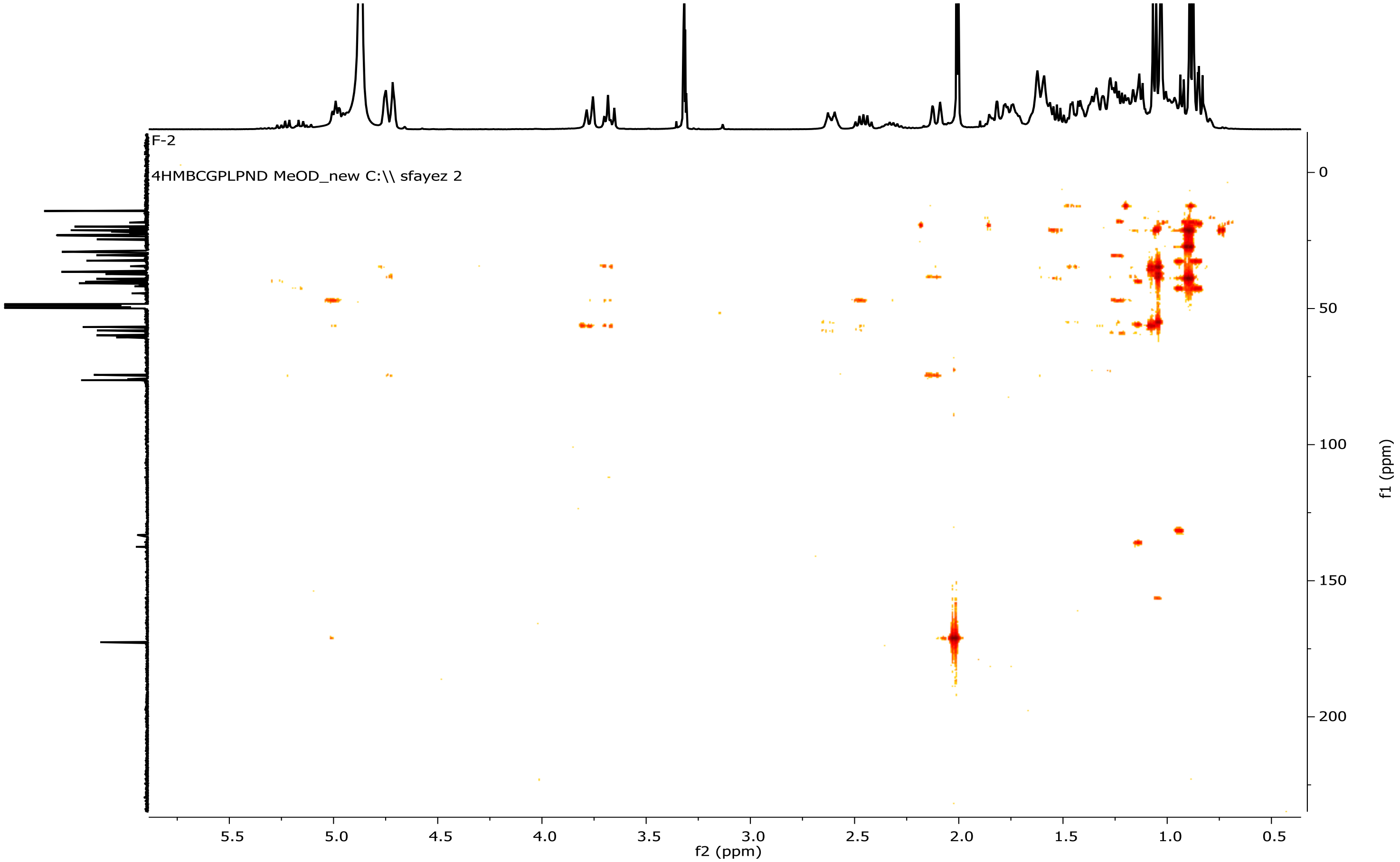

Figure S9. HMBC chart of thalassosterol (**1**, 2β,18-dihydroxy-15α-acetoxy-5,6,7,8-tetrahydroergosterol).

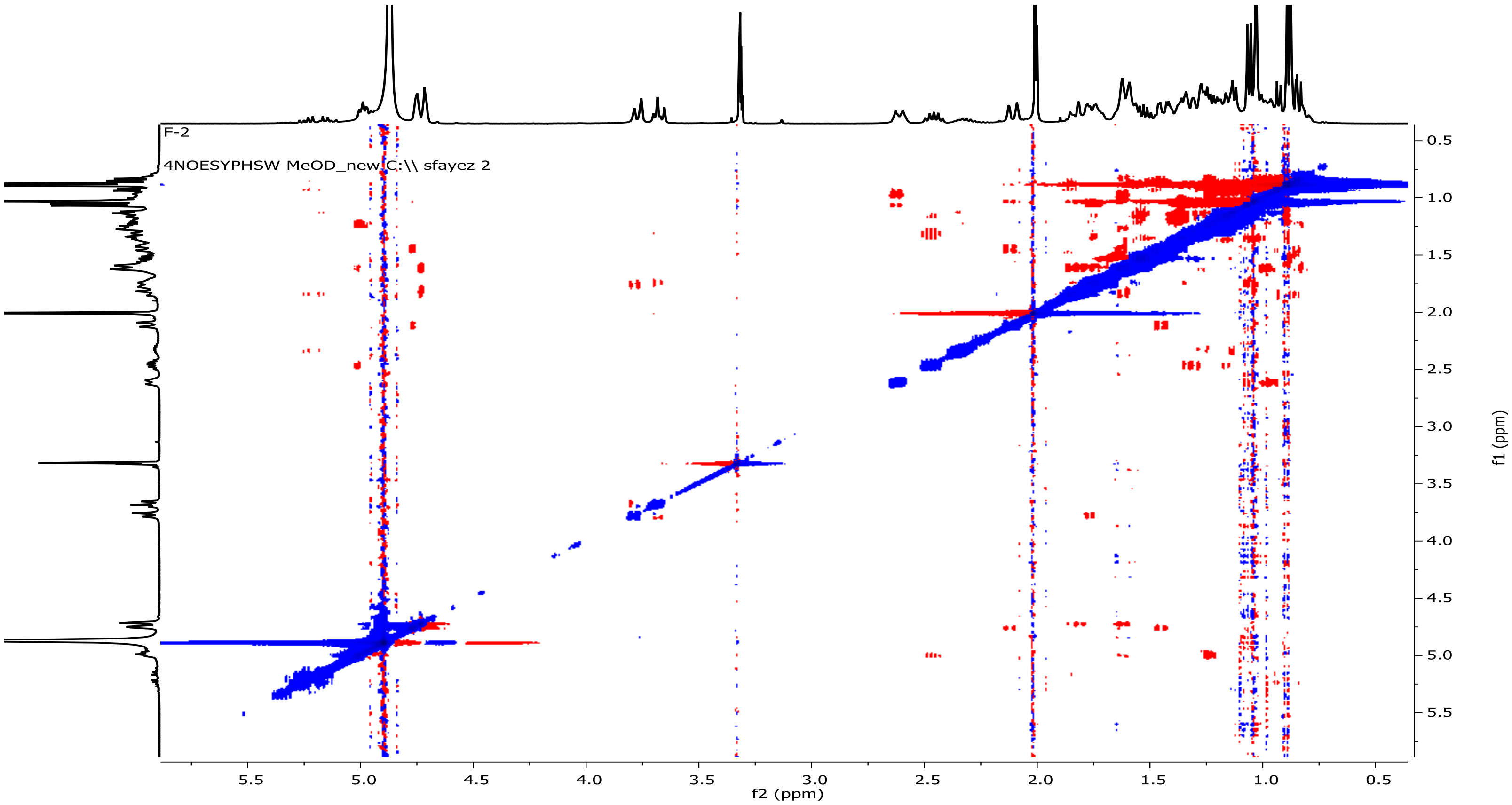

Figure S10. NOESY chart of thalassosterol (**1**, 2*β*,18-dihydroxy-15*α*-acetoxy-5,6,7,8-tetrahydroergosterol).
